# Comprehensive method to detect nitazene analogues and xylazine in wastewater

**DOI:** 10.1007/s11356-025-36425-0

**Published:** 2025-04-22

**Authors:** Emma L. Keller, Brock Peake, Bradley S. Simpson, Jason M. White, Cobus Gerber

**Affiliations:** Clinical and Health Sciences, Health and Biomedical Innovation, University of South Australia, Adelaide, Australia

**Keywords:** Wastewater-based epidemiology, Synthetic opioids, Tranq, Benzimidazole opioids, Novel psychoactive substance, Adulterant

## Abstract

A class of novel synthetic opioids, nitazenes, have infiltrated the illicit drug market worldwide. Their high potency leading to overdose and fatality is a public health concern. Xylazine is a non-opiate sedative typically used for veterinary applications. The drug has recently appeared as an adulterant in the informal drug market, particularly within opioid drugs such as fentanyl and heroin. Determining the extent of use of these drugs is challenging due to low levels consumed. Wastewater analysis has been applied to monitor novel psychoactive substance use at a population level. This study presents the validation of a sensitive analytical method for the detection and quantification of trace levels of nitazenes and xylazine in wastewater. A solid phase extraction method was employed, yielding a concentration factor of 1000-fold. The method was applied to wastewater samples collected in Australia. Five different nitazenes were found near the limit of detection and quantitation levels, with detection frequencies ranging from 3 to 6% of all samples analysed. Xylazine was detected at a higher detection frequency (26% of all samples analysed). The method presented in this study could be used as an early warning system to track the use of xylazine and nitazenes internationally.

**Supplementary Information:**

The online version contains supplementary material available at 10.1007/s11356-025-36425-0.

## Introduction

Nitazenes are potent benzimidazole synthetic opioids and are classed as a novel psychoactive substance (NPS). Originally developed in the 1950 s, the medical use of nitazenes was never approved due to their high potency (Pergolizzi et al. 2023; Ujvary et al. 2021). Depending on the nitazene, they can be several orders of magnitude more potent than morphine (The National Centre for Clinical Research on Emerging Drugs 2024). Fentanyl is often described as a potent opioid (around 100 times more potent than morphine) and has been responsible for thousands of overdose deaths worldwide, most extensively in the USA (Tanz et al. 2024). As some nitazenes are more potent than fentanyl (e.g. etonitazene, protonitazene), their presence in the illicit drug supply is of major public health concern. The first United Nations Office of Drugs and Crime (UNODC) report of nitazenes was in 2019, with increasing reports year on year worldwide (UNODC 2024). The surge of nitazenes identified and reported between 2019 and 2023 coincides with a decrease in the number of new fentanyl analogues being reported (UNODC 2024). During this time, 32 unique nitazenes have been reported to the UNODC up until 2023, with more expected in 2024 (UNODC 2024). New nitazene derivatives are likely to be developed to circumvent scheduling and legislation of these drugs, as well as altering the pharmacological effects, given the history associated with other NPS, such as cannabinoids, cathinones and fentanyl analogues. The use of nitazenes, either intentional or not has resulted in overdoses in Australia, UK, Ireland, Europe and North America and subsequent public warnings about their appearance in the illicit drug market (Killoran et al. 2024; Partridge et al. 2024; Schumann et al. 2023).

Xylazine is a non-opioid sedative that has veterinary applications. Xylazine has been found as an adulterant in illicitly manufactured drugs including non-pharmaceutical fentanyl and/or heroin, prompting widespread alerts and warnings in the USA (Kariisa et al. 2023, National Drug and Early Warning System (2024), U.S. Department of Justice: Drug Enforcement Administration 2022). Xylazine detections in seized material as well as international toxicology reports have risen sharply since 2018 (Cano et al. 2024; Friedman et al. 2022; Galust et al. 2024; Holt et al. 2023; Kacinko et al. 2022). In one study from the USA, xylazine was detected in approximately 80% of drug paraphernalia samples containing fentanyl or fentanyl analogues (Russell et al. 2023). Additionally, none of the participants in this study had intended to purchase xylazine as reported in the questionnaire (Russell et al. 2023). Surveys have also indicated that half of the participants were unaware of xylazine circulating in the illicit drug market, with the majority (73%) indicating they did not want to use the drug (Michaels et al. 2024). There has been an increasing trend in the proportion of illicit fentanyl overdose deaths where xylazine was present, with available data, reporting a change from 2.9% in January 2019 to 10.9% in June 2022 in the USA (Kariisa et al. 2023). Xylazine displays adverse effects, including sedation and nervous system depression, hypotension and skin ulcerations when injected (Edinoff et al. 2024; Hochheimer et al. 2024). Opioid overdose treatment options, such as the administration of naloxone, fail to antagonise the effects of xylazine, making opioid overdoses even more dangerous when xylazine is involved (Edinoff et al. 2024; Love et al. 2023).

Wastewater analysis (WWA) is a tool that has been used to monitor population drug use in near real time and as a measure to indicate and inform policy as an early warning system for the presence of novel psychoactive substances (NPS) (Bade et al. 2019; Jaunay et al. 2024). Xylazine and protonitazene have been detected and measured in wastewater samples collected the USA throughout 2022 (Delcher et al. 2024). Meanwhile, protonitazene was reported in wastewater, also collected in the USA (Bade et al. 2024). To the authors knowledge, there have been no wastewater analysis studies to detect and quantify a range of nitazenes or xylazine in Australian wastewater and the extent of widespread use remains unknown. The aim of this study was to develop and validate an LC–MS/MS analytical method capable of detecting trace amounts of xylazine and nitazenes in wastewater. The extent of use or presence of nitazenes and xylazine in the illicit drug market in Australia is largely unknown. The method was applied to wastewater samples collected from Australian treatment plants.

## Materials and methods

### Chemicals and reagents

Analytical certified reference materials for butonitzene, etonitazene, etodesnitazene, flunitazine, isotonitazene, metonitazene, metodesnitazene, pronitazene, *N*-pyrrolidino etonitazene, *N*-piperidinyl etonitazene, isotonitazene-d3, metonitazene-d3, 4-hydroxyxylazine xylazine and xylazine-d6 were purchased from Novachem (Heidelberg West, Victoria, Australia). Analytical grade acetonitrile, methanol and formic acid were purchased from Chemsupply (Gillman, SA, Australia).

### Wastewater sampling

Wastewater samples from Australian treatment plants were collected as 24-h composite samples representing the daily influent wastewater entering the plant. In total, 180 samples covering both weekday and weekend samples were analysed. Wastewater samples were collected in separate PET bottles containing no preservative, sodium metabisulphite (0.5 g/L) or HCl to acidify the wastewater to pH 2. Sample pre-treatment was conducted by thawing the samples to room temperature followed by filtration under vacuum using glass microfibre filter paper (GF/A 1.6 μm, Whatman, 2 Kent, U.K.). Samples were then processed using solid phase extraction.

### Solid phase extraction

Solid phase extraction was performed using UCT XtracT Clean Screen DAU cartridges (500 mg/6 mL) (UCT, Inc., Bristol, PA, USA). The wastewater sample (100 mL) was adjusted to pH 4.5–5 using 10% v/v acetic acid before loading onto a cartridge, preconditioned with methanol (6 mL) and then sodium acetate buffer (20 mM, pH 5, 6 mL). Samples were extracted under gravity; then, each cartridge was washed successively with sodium acetate buffer (6 mL), 0.1 M acetic acid (2 mL) and methanol (6 mL). The cartridges were eluted using a mixture of dichloromethane: isopropanol and aqueous ammonia (80:16:4, 6 mL). The eluate was dried under nitrogen to approximately 200 µL before 1% HCl in methanol was added to each sample (20 µL) prior to evaporation to dryness. Each sample was reconstituted to a final volume of 100 μL using 0.1% formic acid in methanol (20 μL) and 0.1% formic acid in MilliQ water (80 μL).

Solid phase extraction was also performed using hydrophilic lipophilic balance cartridges (Affinisep® Attract HLB SPE cartridges from PM separations Pty Ltd, Queensland, Australia) as per the manufacturer protocol. Recovery using this HLB stationary phase was poor. Therefore, UCT mixed-mode cartridges were used for all subsequent extractions.

### LC–MS/MS

A Sciex ExionLC coupled to a Sciex 6500 + QTrap (Ontario, Canada), fitted with a TurboSpray IonDrive source, was used for analysis. A Kinetex biphenyl column (150 × 2.1 mm × 1.7 μm) was used for chromatographic separation at a flow rate of 0.3 mL/min. Other column types were tested for performance including C18 and PFP but showed inferior chromatography compared to the biphenyl column. The injection volume was 2 μL. The mobile phases used were 95% water with 5% methanol and 5 mM ammonium formate (solvent A) and 95% methanol with 5% water and 5 mM ammonium formate (solvent B). A linear gradient was used and is reported in Supplementary Table 1. The total run time was 15 min. The ion source parameters were as follows: source temperature, 450 °C; curtain gas, 20; collision gas, medium; ion spray voltage, 5500 V; ion source gas 1 and ion source gas 2, 50. Mass spectrometry analyses were performed in positive mode using multiple reaction monitoring (MRM) with parameters reported in Supplementary Table 2. Confirmation was based on retention time within ± 0.1 min of the reference standard. Further confirmation was performed using high-resolution accurate mass analysis conducted using a Sciex ExionLC system coupled to a Sciex X500R Quadrupole Time-of-Flight (QTOF) mass spectrometer equipped with electrospray ionization (ESI). Exact precursor and fragment ions were confirmed if masses were found within ± 0.02 Da.

### Method validation

The method was validated according to published standard criteria from the European Medicines Agency and the US Department of Health and Human Services Food and Drug Administration and adapted to assess method detection and quantitation limits, analyte loss during filtration, recovery following SPE, matrix effects, precision, accuracy and the stability of both analytes (European Medicines Agency 2011, US Department of Health and Human Services Food and Drug Administration 2018).

#### Standard curve preparation

An eight-point calibration curve was prepared at final in sample concentrations ranging from 2 to 192 ng/L for xylazine and 4-hydroxyxylazine and 0.5–48 ng/L for individual nitazenes. A minimum of six calibration points ± 15% of the nominal concentration were required to pass for a batch to be accepted. The limit of detection (LOD) was determined by analysing matrix matched samples at low concentrations (ranging 0.01 to 1.6 ng/L). The sample that provided a signal to noise ratio of 3:1 was assigned as the LOD and 10:1 for LLOQ. The LOQ was the concentration that provided a signal-to-noise ratio of 10:1 in addition to suitable accuracy ± 20%.

#### Filtration

To assess potential analyte loss during the filtration of raw wastewater, all analytes were spiked into wastewater at two concentrations (5 and 10 ng/L final), then filtered under vacuum. Wastewater without the analyte mixture was also filtered, then spiked with the same amount of analyte mixture post filtration. The experiment was performed in triplicate. The area ratios of both samples were compared and presented as a percentage of the post filtration spiked sample.

#### Recovery and matrix effects

Samples were prepared to determine extraction recovery of each analyte and the matrix effect at three concentrations (1, 5 and 10 ng/L final). The following samples were prepared:

1. Analytes and deuterated analogues in solvent
2. Analytes and deuterated analogues in wastewater followed by SPE
3. Analytes and deuterated analogues in wastewater spiked after SPE
  $$\text{Absolute recovery }(\mathrm{\%})=\frac{\text{Area of analyte set }2}{\text{Area of analyte set }3}$$
  $$\text{Relative recovery }\left(\mathrm{\%}\right)=\frac{\text{Area ratio of set }2}{\text{Area ratio of set }3}$$
  $$\text{Absolute matrix}\left(\mathrm{\%}\right)=\frac{\text{Area of analyte set }3}{\text{Area of analyte set }1}$$
  $$\text{Relative matrix }(\mathrm{\%})=\frac{\text{Area ratio of analyte set }3}{\text{Area ratio of analyte set }1}$$

#### Precision and accuracy

Precision and accuracy were assessed by analysing spiked extracted wastewater samples at four in sample final concentrations (2, 4, 14 and 36 ng/L final for nitazenes and 8, 16, 56 and 144 ng/L final for xylazine and 4-hydroxyxyalzine). Intra-day precision and accuracy were assessed by analysing the samples six times within the same batch on the same day (*n* = 6 for each concentration). Inter-day precision and accuracy were assessed by analysing different samples on three different days (*n* = 18 for each concentration). Precision was determined by calculating the coefficient of variation between each sample by dividing the standard deviation by the average of the area ratio for each sample and expressed as a percentage. A variation of less than 15% was deemed acceptable. Accuracy was determined based on the actual concentration as a percentage of the expected concentration with acceptable values ± 20% of the nominal final in sample concentration for low, medium and high concentrations (4, 14, 36 ng/L, respectively) and ± 15% for the generic LLOQ (2 ng/L).

#### Stability

Stability experiments were performed to monitor analyte degradation at 1, 2, 6, 7 and 14 days at room temperature, 4 °C and − 20 °C. After filtration, wastewater samples were spiked at two concentrations (5 and 10 ng/L). Freshly prepared analyte standards were spiked into three types of wastewater (unpreserved, acidified to pH 2 or sodium metabisulphite 0.5 g/L). On the first day, xylazine-d6 internal standard (final concentration 200 ng/L) was added to triplicate 1 mL aliquots for each type of wastewater and analysed to determine the Day 0 result. The remaining wastewater was stored for 1, 2, 6, 7 and 14 days at either room temperature, 4 °C or − 20 °C. At the end of each time point, internal standard was spiked into each sample and the sample was then analysed. Analyte degradation was assessed using area ratio (peak area of analyte/peak area of internal standard) and reported as a percentage of the area ratio of Day 0 samples.

### Statistical analysis

Integration and analytical processing were performed in the Sciex MultiQuant™ 3.0.3 software. Calculations used for method validation were performed in Microsoft Excel.

## Results and discussion

**Fig. 1:**
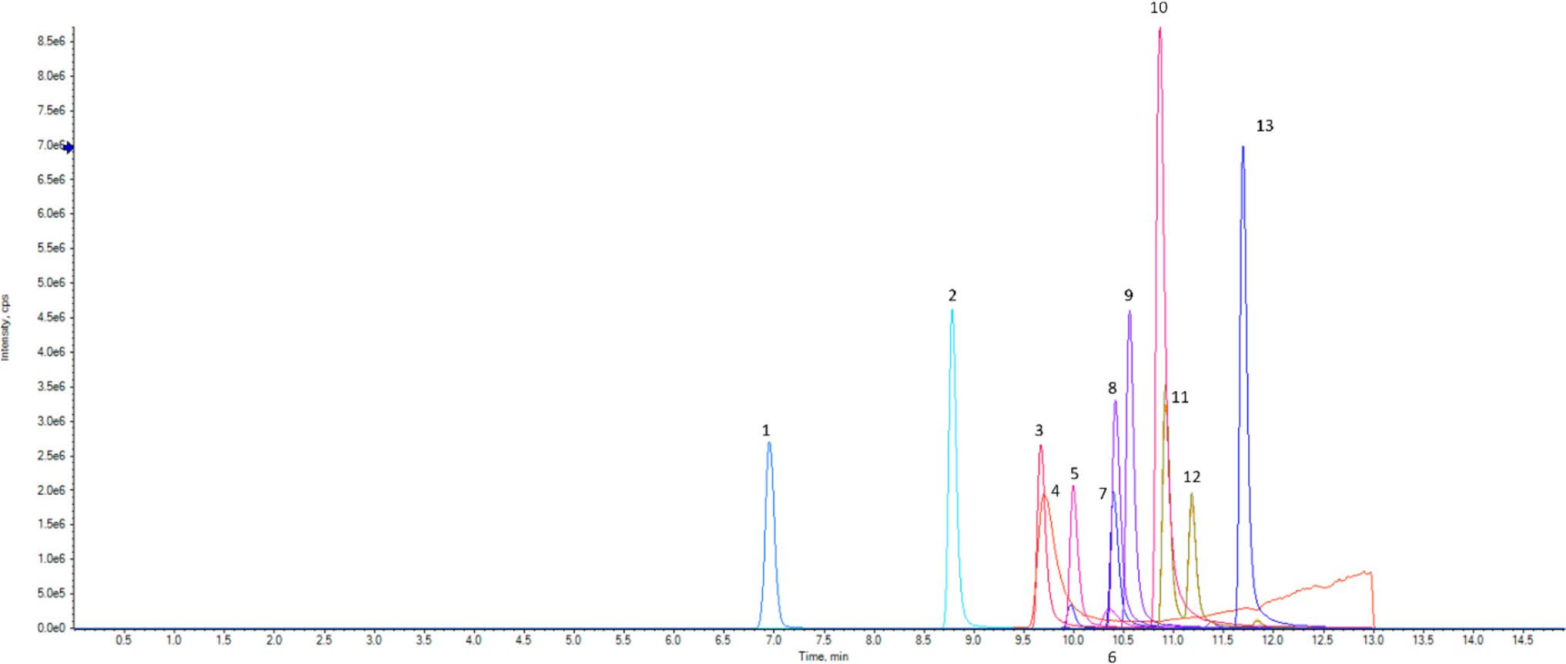
Extracted ion chromatogram of the nitazenes and xylazine included in the analytical method. (1) 4-Hydroxyxylazine, (2) xylazine, (3) flunitazene, (4) metodesnitazene, (5) metonitazene, (6) etodesnitazene, (7) clonitazene, (8) *N*-pyrrolidino etonitazene, (9) etonitazene, (10) *N*-piperidinyl etonitazene, (11) isotonitazene, (12) protonitazene, and (13) butonitazene

An analytical method capable of separating and detecting 11 nitazenes, xylazine and 4-hydroxyxylazine was developed and validated in this study. The chromatographic separation of nitazene analogues is critical to distinguish between isomers (i.e. isotonitazene and protonitazene). The separation of all the analytes included in the method is shown in Fig. 1.

All analyte calibration plots showed acceptable linearity over a concentration range of 0.5–48 ng/L (Table 1). Calibration plots prepared at low concentrations to determine the LOD, LLOQ and LOQ resulted in below 1 ng/L sensitivity ranges. All analytes included in the method showed minimal filtration loss (between 79 and 108% recovery of the analyte after filtration). Recovery and matrix effects were assessed for both classes (Supplementary Table 3).

**Table 1:** Linearity, detection and quantitation limits of the method for nitazenes and xylazine

Analyte	Linearity*	LOD (ng/L)	LLOQ (ng/L)	LOQ (ng/L)	Filtration recovery (%)
Butonitazene^1^	0.9939	0.1	0.4	0.6	107 ± 5
Clonitazene^2^	0.9961	0.15	0.15	0.4	83 ± 4
Etodesnitazene^1^	0.9903	0.075	0.1	0.4	104 ± 9
Etonitazene^2^	0.9938	0.1	0.2	0.4	90 ± 6
Flunitazene^1^	0.9946	0.4	0.4	0.8	79 ± 5
Isotonitazene^1^	0.9946	0.15	0.4	0.4	96 ± 5
Metodesnitazene^2^	0.9856	0.2	0.3	0.6	108 ± 7
Metonitazene^2^	0.9940	0.2	0.3	0.6	81 ± 6
Protonitazene^1^	0.9956	0.2	0.4	0.6	95 ± 15
*N*-pyrrolidino etonitazene^1^	0.9934	0.2	0.4	0.8	85 ± 8
*N-*piperidinyl etonitazene^1^	0.9926	0.075	0.15	0.25	101 ± 6
Xylazine^3^	0.9965	0.05	0.075	0.1	94 ± 5
4-Hydroxyxylazine^3^	0.9960	0.15	0.3	0.4	89 ± 1

*Abbreviations*: LOD: limit of detection—concentration with signal to noise ratio of intensity 3:1, LLOQ: lower limit of quantification—concentration with signal to noise intensity of 10:1, LOQ: limit of quantification—concentration that provides signal to noise ratio of 10:1 and passes within ± 20% of nominal concentration in wastewater

^1^Isotonitazene-d6

^2^Metonitazene-d3

^3^Xylazine-d3, internal standard used for correction

*Linearity based on concentration ranges of 1–100 ng/L for the nitazenes and 2–192 ng/L for xylazine and 4-hydroxyxylazine

All analytes were sufficiently recovered following solid phase extraction when an internal standard was used to correct for potential loss (Supplementary Table 3). Matrix effects were variable with up to 78% absolute matrix suppression observed. The matrix suppression was sufficiently corrected for when using a nitazene internal standard for all analytes except butonitazene. The two nitazene internal standards adopted in this study (isotonitazene-d3, metonitazene-d3) were unable to sufficiently correct butonitazene matrix suppression, with up to 35% relative matrix suppression observed (Supplementary Table 3).

The method performance parameters of precision and accuracy was acceptable for both intra- and inter-day (Supplementary Table 4). The method variability was minimal, with precision values equal to or less than 13% for intra-day or inter-day analysis for most analytes. The method showed suitable accuracy, with measured concentrations within ± 15% of the nominal concentration. Accuracy and precision did not perform within the guidelines for butonitazene and protonitazene. This may be due to the use of surrogate internal standards and higher matrix suppression observed for these analytes (Supplementary Table 3). If quantification of these analytes is required, a matched internal standard is required, or a calibration curve prepared in wastewater matrix. The limit of detection and quantification of the method was performed by preparing matrix matched calibration curves at low levels; hence, accuracy was within ± 20%.

**Fig. 2:**
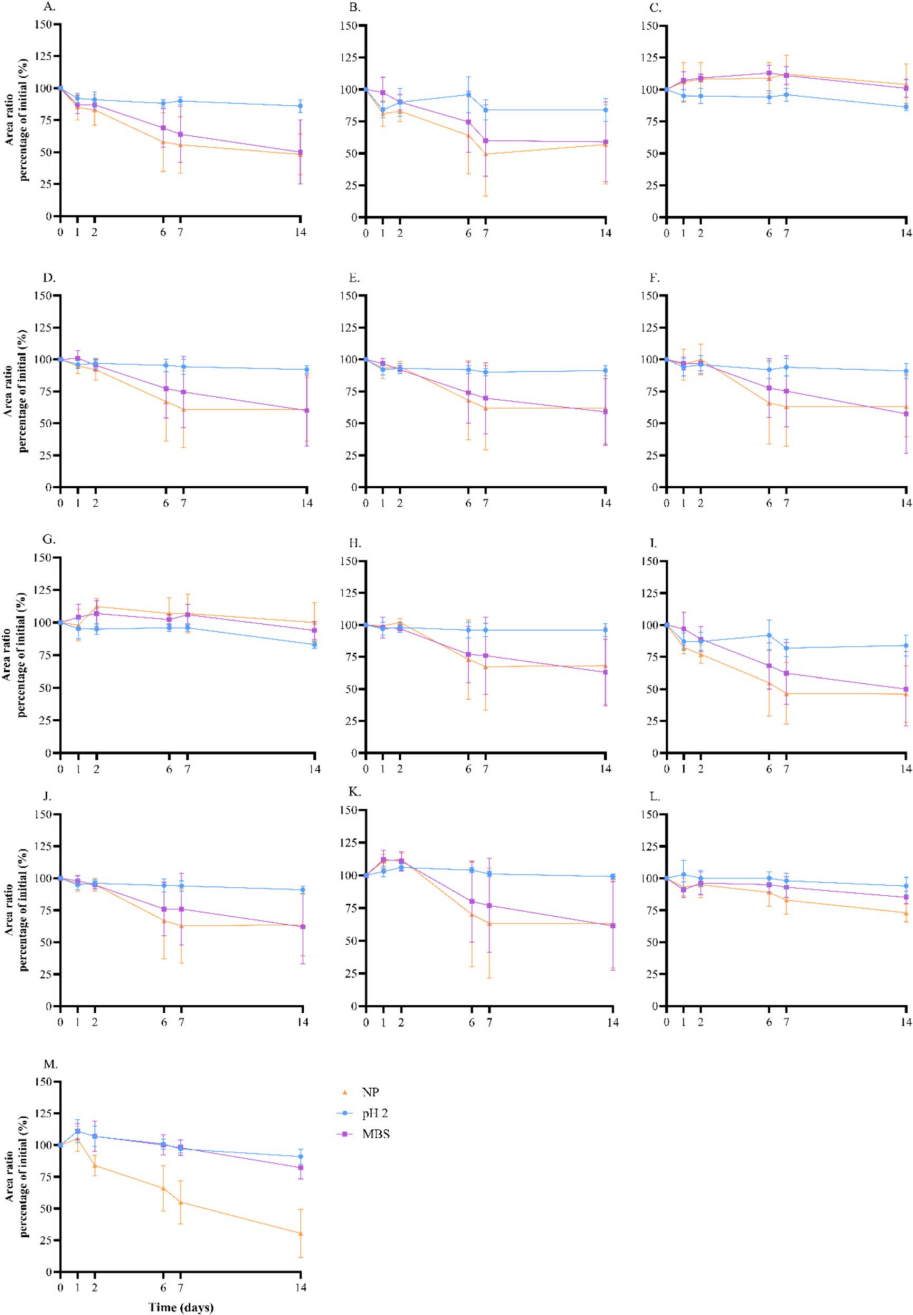
Stability for analytes included in the method stored at room temperature. (**A**) Butonitazene, (**B**) clonitazene, (**C**) etodesnitazene, (**D**) etonitazene, (**E**) flunitazene, (**F**) isotonitazene, (**G**) metodesnitazene, (**H**) metonitazene, (**I**) protonitazene, (**J**) *N*-piperidinyl etonitazene, (**K**) *N*-pyrrolidino etonitazene, (**L**) xylazine, and (**M**) 4-hydroxyxylazine

The stability of the analytes in wastewater was assessed over 14 days at different room temperature (Fig. 2). Degradation was observed when wastewater was unpreserved or in the presence of sodium metabisulphite at room temperature for most nitazenes and 4-hydroxyxylazine (Fig. 2). Preservation with sodium metabisulphite or acidifying at pH2 and storing samples at 4 °C or − 20 °C resulted in minimal degradation of the analytes over 14 days (Supplementary Tables 5 and 6). The acidification or addition of sodium metabisulphite is standard practice, with wastewater samples stored at − 20 °C. Therefore, minimal degradation of the target analytes would be expected when following standardised storage protocols.

The method was applied to wastewater collected from Australian wastewater treatment plants in August 2024. Three days (one weekday and both weekend days) were analysed from a total of 60 sites equating to 180 samples. Detections of nitazenes was low, with 2% of the total samples analysed containing near LOD amounts of butonitazene, etonitazene and etodesnitazene (Table 2). Slightly higher detection frequencies were recorded for *N*-pyrrolidino etonitazene (3% of all samples) and *N*-piperidinyl etonitazene (6%). Xylazine was found across many sites, with 26% of all samples containing above LOQ levels in wastewater ranging from LOQ (0.1 ng/L) to 207 ng/L. The xylazine detections were confirmed with accurate mass using ToF.

**Table 2:** Detection frequency of nitazenes and xylazine in Australian wastewater

Analyte	Frequency of positive sample detections (*N* = 180 samples) (%)
Butonitazene	2
Etonitazene	2
Etodesnitazene	2
*N*-Piperidinyl etonitazene	6
*N*-Pyrrolidino etonitazene	3
Xylazine	26

Current methods to detect nitazenes in various biological and environmental samples are being developed, including lateral flow immunoassay test strips, gas and liquid chromatography mass spectrometry. Immunoassays and gas chromatography may lack the selectivity or sensitivity required to detect these analytes in wastewater as the substances are consumed sporadically and in low quantities. While direct injection has been used successfully to detect common drugs in wastewater, solid phase extraction results in preconcentration of the wastewater sample and reduces interferences to enable low detection limits. The method developed in this study has the flexibility to be updated frequently as new structural derivatives enter the illicit drug market. This study has shown that a range of nitazenes and xylazine can be detected in influent wastewater samples in Australia. The method presented in this study could be used as an early warning system to track the use of xylazine and nitazenes internationally.

## Supplementary Information

Below is the link to the electronic supplementary material.

Supplementary file1 (DOCX 54 KB)

## Data Availability

Data will be made available upon request.
