# Supplementary material for: Comprehensive method to detect nitazene analogues and xylazine in wastewater
Source: Environ Sci Pollut Res Int. 2025 Apr 22;32(57):30863–70. doi: 10.1007/s11356-025-36425-0 (PMC12811310; doi:10.1007/s11356-025-36425-0)
Supplement: Supplementary file 1 — Supplementary file1 (DOCX 54 KB) [file 11356_2025_36425_MOESM1_ESM.docx]

**Comprehensive method to detect nitazene analogues and xylazine in wastewater**

Emma L. Keller^1a^, Brock Peake^1a^, Bradley S. Simpson^1^, Jason M. White^1^, and Cobus Gerber^1*^

^1^Clinical and Health Sciences, Health and Biomedical Innovation, University of South Australia, Australia.

^a^Co-first authors

*Corresponding author

**Supplementary Material:**

**Supplementary Table 1. Liquid chromatography gradient program adopted for the method.**

| **Time (min)** | **A. Conc** | **B. Conc** |
| --- | --- | --- |
| 0 | 90 | 10 |
| 2 | 90 | 10 |
| 12 | 0 | 100 |
| 13 | 0 | 100 |
| 13.1 | 90 | 10 |

**Supplementary Table 2. Mass spectrometry parameters for analytes included in the method.**

|  | **Analyte** | **Precursor ion (Q1)** | **Product ions (Q3)** | **Collision energy (V)** | **Declustering potential (DP)** | **Collision cell exit potential (CXP)** |
| --- | --- | --- | --- | --- | --- | --- |
| A | Butonitazene | 425.2 | 100.1/72.1 | 26 | 75 | 17 |
| B | Clonitazene | 387.1 | 100.1/72.1 | 43 | 46 | 16 |
| C | Etodesnitazene | 353.1 | 100.1/72.1 | 22 | 75 | 17 |
| D | Etonitazene | 397.4 | 100.1/72.1 | 44 | 45 | 15 |
| E | Flunitazene | 371.3 | 100.1/72.1 | 41 | 42 | 15 |
| F | Isotonitazene | 411.1 | 100.1/72.1 | 25 | 75 | 17 |
| G | Metodesnitazene | 338.4 | 100.1/72.1 | 25 | 75 | 17 |
| H | Metonitazene | 383.3 | 100.1/72.1 | 30 | 65 | 23 |
| I | Protonitazene | 411.5 | 100.1/72.1 | 26 | 75 | 17 |
| J | *N*-pyrrolidino etonitazene | 395.2 | 98.0 | 26 | 75 | 17 |
| K | *N-*piperidinyl etonitazene | 409.2 | 112.1 | 28 | 75 | 17 |
| L | Xylazine | 221.1 | 164.1/90.1 | 35 | 75 | 17 |
| M | 4-hydroxyxylazine | 237.1 | 180.1/163.1 | 35 | 75 | 17 |
|  | Isotonitazene-d6 | 417.1 | 100.3 | 24 | 60 | 10 |
|  | Metonitazene-d3 | 386.3 | 100.3 | 31 | 75 | 17 |
|  | Xylazine-d6 | 227.1 | 170.1 | 35 | 75 | 17 |

Entrance potential = 10 volts for all analytes.

**Supplementary Table 3. Recovery of each analyte after solid phase extraction and wastewater matrix effects**

| **Analyte** | **Absolute recovery (%)** | | |  | **Relative recovery (%)** | | | **Absolute matrix effects (%)** | | | **Relative matrix effects (%)** | | |
| --- | --- | --- | --- | --- | --- | --- | --- | --- | --- | --- | --- | --- | --- |
|  | L | M | H | | L | M | H | L | M | H | L | M | H |
| Butonitazene^1^ | 77 ± 32 | 68 ± 25 | 77 ± 20 | | 90 ± 13 | 93 ± 20 | 89 ± 7 | 26 ± 13 | 32 ± 10 | 22 ± 4 | 107 ± 26 | 65 ± 8 | 66 ± 5 |
| Clonitazene^2^ | 79 ± 8 | 74 ± 12 | 86 ± 15 | | 92 ± 9 | 92 ± 11 | 85 ± 8 | 36 ± 7 | 38 ± 8 | 26 ± 2 | 109 ± 11 | 101 ± 6 | 77 ± 1 |
| Etodesnitazene^1^ | 70 ± 20 | 88 ± 7 | 90 ± 17 | | 86 ± 16 | 125 ± 11 | 105 ± 2 | 79 ± 20 | 70 ± 9 | 56 ± 4 | 141 ± 25 | 149 ± 20 | 163 ± 17 |
| Etonitazene^2^ | 78 ± 6 | 78 ± 13 | 92 ± 13 | | 91 ± 5 | 95 ± 8 | 90 ± 4 | 45 ± 13 | 44 ± 8 | 33 ± 2 | 110 ± 6 | 118 ± 2 | 95 ± 5 |
| Flunitazene^1^ | 82 ± 6 | 81 ± 12 | 99 ± 8 | | 102 ± 6 | 99 ± 2 | 117 ± 16 | 43 ± 11 | 37 ± 6 | 32 ± 1 | 98 ± 6 | 100 ± 1 | 97 ± 11 |
| Isotonitazene^1^ | 76 ± 6 | 71 ± 10 | 86 ± 15 | | 95 ± 3 | 100 ± 5 | 100 ± 5 | 40 ± 13 | 50 ± 12 | 32 ± 2 | 106 ± 3 | 105 ± 1 | 100 ± 8 |
| Metodesnitazene^2^ | 83 ± 5 | 82 ± 10 | 94 ± 8 | | 96 ± 6 | 101 ± 8 | 92 ± 3 | 99 ± 42 | 59 ± 8 | 63 ± 9 | 104 ± 6 | 160 ± 5 | 164 ± 25 |
| Metonitazene^2^ | 83 ± 2 | 80 ± 9 | 102 ± 11 | | 97 ± 8 | 98 ± 3 | 101 ± 1 | 51 ± 10 | 37 ± 6 | 35 ± 1 | 104 ± 9 | 101 ± 2 | 103 ± 7 |
| Protonitazene^1^ | 76 ± 12 | 69 ± 17 | 81 ± 17 | | 95 ± 14 | 95 ± 8 | 94 ± 4 | 36 ± 15 | 45 ± 12 | 28 ± 3 | 107 ± 15 | 93 ± 8 | 86 ± 5 |
| *N-*piperidinyl etonitazene^1^ | 79 ± 14 | 74 ± 13 | 85 ± 17 | | 97 ± 11 | 91 ± 8 | 99 ± 0 | 37 ± 14 | 74 ± 13 | 30 ± 4 | 104 ± 12 | 103 ± 8 | 91 ± 5 |
| *N*-pyrrolidino etonitazene^1^ | 83 ± 6 | 74 ± 15 | 92 ± 17 | | 104 ± 8 | 103 ± 5 | 106 ± 3 | 42 ± 14 | 39 ± 9 | 30 ± 2 | 97 ± 7 | 82 ± 2 | 93 ± 6 |
| Xylazine^3*^ | 91 ± 6 | 88 ± 17 | 105 ± 4 | | 96 ± 3 | 101 ± 6 | 103 ± 2 | 120 ± 19 | 55 ± 6 | 72 ± 6 | 104 ± 4 | 104 ± 3 | 92 ± 7 |
| 4-hydroxyxylazine^3*^ | 107 ± 9 | 91 ± 14 | 119 ± 3 | | 113 ± 5 | 105 ± 4 | 117 ± 4 | 122 ± 13 | 45 ± 3 | 71 ± 7 | 89 ± 4 | 86 ± 2 | 91 ± 8 |

^1^: Isotonitazene-d6, ^2^: Metonitazene-d3, ^3^: Xylazine-d3, internal standard used for correction. Recovery expressed as percentage is the amount of analyte remaining after extraction. Matrix effects expressed as a percentage indicates the signal response in wastewater compared to a solvent sample. Values under 100 indicate matrix suppression and values above 100 indicate matrix enhancement.

**Supplementary Table 4. Precision and accuracy of the analytical method.**

| **Analyte** | **Precision (CV %)** | | | | | | | | **Accuracy (%)** | | | | | | | |
| --- | --- | --- | --- | --- | --- | --- | --- | --- | --- | --- | --- | --- | --- | --- | --- | --- |
|  | LOQ |  | Low | | Med |  | High | | LOQ |  | Low | | Med |  | High | |
|  | Intra-day | Inter-day | Intra-day | Inter-day | Intra-day | Inter-day | Intra-day | Inter-day | Intra-day | Inter-day | Intra-day | Inter-day | Intra-day | Inter-day | Intra-day | Inter-day |
| Butonitazene | 6 | 15 | 12 | 37 | 3 | 39 | 6 | 25 | 39 ± 6 | 34 ± 5 | 71 ± 9 | 48 ± 18 | 70 ± 2 | 46 ± 18 | 56 ± 3 | 42 ± 10 |
| Clonitazene | 13 | 11 | 3 | 10 | 2 | 5 | 4 | 3 | 114 ± 15 | 111 ± 12 | 96 ± 3 | 94 ± 9 | 86 ± 2 | 86 ± 4 | 82 ± 3 | 83 ± 3 |
| Etodesnitazene | 10 | 12 | 11 | 13 | 5 | 4 | 6 | 5 | 105 ± 11 | 100 ± 12 | 101 ± 11 | 104 ± 14 | 113 ± 6 | 115 ± 5 | 112 ± 7 | 113 ± 6 |
| Etonitazene | 3 | 4 | 2 | 8 | 3 | 5 | 3 | 4 | 113 ± 3 | 113 ± 4 | 111 ± 2 | 101 ± 8 | 112 ± 3 | 111 ± 6 | 115 ± 3 ± | 116 ± 5 |
| Flunitazene | 3 | 4 | 4 | 3 | 2 | 3 | 4 | 2 | 85 ± 2 | 89 ± 4 | 90 ± 4 | 89 ± 3 | 94 ± 2 | 92 ± 2 | 90 ± 3 | 90 ± 2 |
| Isotonitazene | 2 | 3 | 2 | 2 | 3 | 4 | 1 | 1 | 99 ± 2 | 101 ± 3 | 102 ± 2 | 101 ± 2 | 106 ± 3 | 105 ± 4 | 103 ± 1 | 104 ± 2 |
| Metodesnitazene | 8 | 8 | 6 | 6 | 4 | 6 | 3 | 7 | 108 ± 8 | 102 ± 8 | 95 ± 6 | 100 ± 6 | 109 ± 4 | 114 ± 6 | 103 ± 3 | 112 ± 8 |
| Metonitazene | 3 | 6 | 2 | 3 | 1 | 3 | 2 | 4 | 100 ± 3 | 107 ± 7 | 100 ± 2 | 103 ± 3 | 103 ± 1 | 105 ± 3 | 98 ± 2 | 103 ± 4 |
| Protonitazene | 4 | 20 | 14 | 31 | 3 | 6 | 4 | 6 | 38 ± 2 | 48 ± 10 | 60 ± 2 | 62 ± 4 | 59 ± 2 | 60 ± 4 | 40 ± 21 | 54 ± 17 |
| N-piperidinyl etonitazene | 3 | 4 | 3 | 5 | 2 | 8 | 1 | 4 | 86 ± 2 | 83 ± 3 | 93 ± 3 | 88 ± 5 | 94 ± 2 | 86 ± 6 | 91 ± 1 | 87 ± 3 |
| N-pyrrolidino etonitazene | 1 | 12 | 2 | 7 | 2 | 5 | 2 | 8 | 98 ± 1 | 90 ± 11 | 83 ± 1 | 86 ± 6 | 87 ± 2 | 90 ± 4 | 82 ± 1 | 91 ± 7 |
| Xylazine | 2 | 3 | 1 | 2 | 3 | 2 | 2 | 5 | 103 ± 2 | 108 ± 3 | 107 ± 1 | 109 ± 2 | 116 ± 3 | 115 ± 2 | 99 ± 2 | 106 ± 5 |
| 4-hydroxynitazene | 2 | 2 | 2 | 3 | 1 | 3 | 0 | 4 | 96 ± 2 | 95 ± 2 | 96 ± 2 | 94 ± 3 | 105 ± 1 | 102 ± 3 | 105 ± 0 | 100 ± 4 |

**Supplementary Table 5. Stability of analytes at 4 °C for 14 days**

|  | **NP** | | | | | **pH 2** | | | | | | **MBS** | | | | |
| --- | --- | --- | --- | --- | --- | --- | --- | --- | --- | --- | --- | --- | --- | --- | --- | --- |
| **Analyte** | **1** | **2** | **6** | **7** | **14** | **1** | **2** | **6** | **7** | **14** | **1** | | **2** | **6** | **7** | **14** |
| Butonitazene | 82 ± 9 | 79 ± 11 | 75 ± 8 | 71 ± 9 | 68 ± 8 | 90 ± 4 | 90 ± 4 | 91 ± 3 | 87 ± 3 | 84 ± 3 | 85 ± 6 | | 83 ± 7 | 76 ± 12 | 72 ± 3 | 76 ± 8 |
| Clonitazene | 92 ± 17 | 85 ± 14 | 91 ± 10 | 75 ± 6 | 77 ± 9 | 92 ± 10 | 86 ± 10 | 88 ± 12 | 86 ± 9 | 85 ± 9 | 101 ± 12 | | 89 ± 8 | 92 ± 11 | 82 ± 13 | 92 ± 10 |
| Etodesnitazene | 111 ± 15 | 112 ± 14 | 115 ± 15 | 113 ± 15 | 103 ± 14 | 98 ± 6 | 96 ± 7 | 97 ± 6 | 97 ± 3 | 88 ± 6 | 113 ± 8 | | 112 ± 4 | 112 ± 5 | 114 ± 5 | 107 ± 5 |
| Etonitazene | 97 ± 6 | 90 ± 6 | 95 ± 6 | 92 ± 6 | 89 ± 9 | 97 ± 4 | 88 ± 6 | 96 ± 3 | 95 ± 4 | 92 ± 3 | 101 ± 4 | | 97 ± 5 | 97 ± 3 | 96 ± 3 | 97 ± 2 |
| Flunitazene | 95 ± 7 | 98 ± 6 | 97 ± 6 | 93 ± 6 | 94 ± 9 | 92 ± 5 | 95 ± 4 | 94 ± 3 | 92 ± 4 | 94 ± 3 | 96 ± 3 | | 97 ± 4 | 94 ± 3 | 92 ± 4 | 96 ± 3 |
| Isotonitazene | 99 ± 12 | 99 ± 15 | 94 ± 14 | 87 ± 12 | 85 ± 11 | 97 ± 10 | 96 ± 7 | 93 ± 7 | 87 ± 6 | 84 ± 5 | 99 ± 3 | | 98 ± 4 | 92 ± 6 | 89 ± 6 | 91 ± 4 |
| Metodesnitazene | 110 ± 13 | 112 ± 10 | 107 ± 14 | 111 ± 11 | 100 ± 15 | 97 ± 5 | 94 ± 4 | 96 ± 3 | 95 ± 4 | 86 ± 4 | 105 ± 6 | | 109 ± 11 | 101 ± 7 | 103 ± 5 | 102 ± 3 |
| Metonitazene | 103 ± 5 | 101 ± 5 | 101 ± 5 | 100 ± 5 | 98 ± 7 | 98 ± 5 | 97 ± 5 | 98 ± 5 | 98 ± 6 | 96 ± 4 | 100 ± 4 | | 101 ± 3 | 98 ± 5 | 97 ± 5 | 100 ± 6 |
| Protonitazene | 78 ± 5 | 77 ± 7 | 75 ± 4 | 69 ± 8 | 69 ± 8 | 87 ± 6 | 84 ± 8 | 84 ± 8 | 78 ± 5 | 78 ± 6 | 93 ± 10 | | 86 ± 12 | 86 ± 9 | 78 ± 2 | 79 ± 7 |
| *N*-pyrrolidino etonitazene | 116 ± 7 | 117 ± 9 | 115 ± 5 | 108 ± 7 | 108 ± 9 | 104 ± 4 | 107 ± 5 | 106 ± 2 | 102 ± 6 | 103 ± 3 | 116 ± 4 | | 113 ± 7 | 111 ± 8 | 106 ± 9 | 111 ± 6 |
| *N-*piperidinyl etonitazene | 97 ± 7 | 95 ± 5 | 90 ± 7 | 90 ± 9 | 87 ± 9 | 97 ± 3 | 94 ± 4 | 92 ± 2 | 93 ± 4 | 90 ± 4 | 99 ± 3 | | 98 ± 3 | 94 ± 3 | 91 ± 4 | 94 ± 2 |
| Xylazine | 100 ± 3 | 108 ± 9 | 99 ± 9 | 97 ± 5 | 90 ± 9 | 98 ± 10 | 101 ± 8 | 101 ± 5 | 99 ± 9 | 97 ± 8 | 90 ± 5 | | 96 ± 6 | 97 ± 6 | 96 ± 7 | 97 ± 5 |
| 4-hydroxy xylazine | 106 ± 6 | 93 ± 11 | 102 ± 5 | 94 ± 8 | 75 ± 6 | 109 ± 6 | 109 ± 8 | 100 ± 2 | 102 ± 3 | 93 ± 4 | 113 ± 12 | | 104 ± 9 | 104 ± 7 | 103 ± 6 | 95 ± 9 |

**Supplementary Table 6. Stability of analytes at -20 °C for 14 days**

|  | **NP** | | | | | **pH 2** | | | | | | **MBS** | | | | |
| --- | --- | --- | --- | --- | --- | --- | --- | --- | --- | --- | --- | --- | --- | --- | --- | --- |
| **Analyte** | **1** | **2** | **6** | **7** | **14** | **1** | **2** | **6** | **7** | **14** | **1** | | **2** | **6** | **7** | **14** |
| Butonitazene | 82 ± 8 | 77 ± 10 | 73 ± 9 | 78 ± 10 | 71 ± 6 | 95 ± 5 | 87 ± 5 | 88 ± 7 | 88 ± 5 | 83 ± 4 | 86 ± 5 | | 84 ± 5 | 80 ± 8 | 76 ± 6 | 76 ± 5 |
| Clonitazene | 87 ± 10 | 84 ± 5 | 81 ± 17 | 77 ± 9 | 88 ± 10 | 89 ± 6 | 84 ± 6 | 83 ± 6 | 85 ± 8 | 85 ± 4 | 92 ± 7 | | 93 ± 13 | 90 ± 13 | 99 ± 18 | 88 ± 9 |
| Etodesnitazene | 112 ± 11 | 113 ± 13 | 114 ± 15 | 108 ± 16 | 108 ± 14 | 99 ± 5 | 95 ± 6 | 96 ± 5 | 91 ± 3 | 88 ± 5 | 114 ± 2 | | 113 ± 5 | 115 ± 6 | 104 ± 5 | 104 ± 8 |
| Etonitazene | 97 ± 3 | 94 ± 6 | 94 ± 7 | 90 ± 5 | 93 ± 5 | 98 ± 4 | 91 ± 5 | 93 ± 5 | 95 ± 9 | 92 ± 5 | 103 ± 4 | | 98 ± 2 | 98 ± 2 | 94 ± 2 | 97 ± 8 |
| Flunitazene | 95 ± 4 | 98 ± 6 | 97 ± 5 | 89 ± 5 | 99 ± 5 | 95 ± 6 | 94 ± 6 | 93 ± 6 | 91 ± 4 | 92 ± 6 | 97 ± 4 | | 97 ± 4 | 97 ± 4 | 90 ± 3 | 95 ± 7 |
| Isotonitazene | 100 ± 8 | 98 ± 12 | 92 ± 13 | 78 ± 11 | 86 ± 10 | 98 ± 8 | 93 ± 11 | 87 ± 8 | 73 ± 7 | 78 ± 10 | 103 ± 5 | | 94 ± 5 | 91 ± 4 | 80 ± 4 | 87 ± 8 |
| Metodesnitazene | 104 ± 10 | 114 ± 11 | 112 ± 9 | 90 ± 7 | 103 ± 10 | 98 ± 3 | 95 ± 6 | 98 ± 8 | 82 ± 5 | 88 ± 5 | 101 ± 6 | | 110 ± 9 | 104 ± 11 | 88 ± 4 | 96 ± 7 |
| Metonitazene | 102 ± 4 | 103 ± 3 | 103 ± 3 | 98 ± 5 | 102 ± 3 | 97 ± 4 | 97 ± 3 | 97 ± 6 | 98 ± 4 | 96 ± 7 | 101 ± 4 | | 99 ± 4 | 103 ± 6 | 97 ± 6 | 100 ± 9 |
| Protonitazene | 80 ± 6 | 74 ± 6 | 81 ± 6 | 74 ± 7 | 77 ± 5 | 91 ± 6 | 85 ± 7 | 86 ± 8 | 81 ± 6 | 81 ± 7 | 87 ± 10 | | 86 ± 12 | 86 ± 9 | 78 ± 2 | 79 ± 7 |
| *N*-pyrrolidino etonitazene | 116 ± 5 | 118 ± 6 | 115 ± 5 | 107 ± 4 | 114 ± 7 | 109 ± 2 | 105 ± 4 | 104 ± 4 | 102 ± 6 | 101 ± 4 | 118 ± 6 | | 112 ± 3 | 114 ± 7 | 108 ± 7 | 113 ± 10 |
| *N-*piperidinyl etonitazene | 96 ± 5 | 93 ± 6 | 92 ± 7 | 86 ± 5 | 92 ± 7 | 97 ± 2 | 90 ± 4 | 94 ± 7 | 91 ± 4 | 92 ± 4 | 98 ± 3 | | 95 ± 4 | 98 ± 2 | 89 ± 2 | 94 ± 7 |
| Xylazine | 97 ± 8 | 105 ± 7 | 102 ± 5 | 97 ± 6 | 96 ± 6 | 95 ± 6 | 103 ± 7 | 102 ± 10 | 101 ± 8 | 99 ± 10 | 90 ± 6 | | 99 ± 8 | 97 ± 8 | 94 ± 7 | 94 ± 9 |
| 4-hydroxy xylazine | 101 ± 11 | 85 ± 8 | 91 ± 4 | 76 ± 6 | 83 ± 10 | 106 ± 3 | 107 ± 4 | 98 ± 6 | 98 ± 8 | 94 ± 9 | 108 ± 10 | | 109 ± 7 | 102 ± 7 | 93 ± 3 | 94 ± 7 |
